# Encapsulation of ropivacaine in a combined (donor-acceptor, ionic-gradient) liposomal system promotes extended anesthesia time

**DOI:** 10.1371/journal.pone.0185828

**Published:** 2017-10-05

**Authors:** Camila Morais Gonçalves da Silva, Michelle Franz-Montan, Cíntia Elisabeth Gomez Limia, Lígia Nunes de Morais Ribeiro, Mário Antônio Braga, Viviane Aparecida Guilherme, Camila Batista da Silva, Bruna Renata Casadei, Cíntia Maria Saia Cereda, Eneida de Paula

**Affiliations:** 1 Department of Biochemistry and Tissue Biology, Institute of Biology, University of Campinas–UNICAMP, Campinas, SP, Brazil; 2 Department of Physiological Sciences, Piracicaba Dental School, University of Campinas, UNICAMP, Piracicaba, SP, Brazil; Massachusetts General Hospital, UNITED STATES

## Abstract

Ropivacaine is a local anesthetic with similar potency but lower systemic toxicity than bupivacaine, the most commonly used spinal anesthetic. The present study concerns the development of a combined drug delivery system for ropivacaine, comprised of two types of liposomes: donor multivesicular vesicles containing 250 mM (NH_4_)_2_SO_4_ plus the anesthetic, and acceptor large unilamellar vesicles with internal pH of 5.5. Both kinds of liposomes were composed of hydrogenated soy-phosphatidylcholine:cholesterol (2:1 mol%) and were prepared at pH 7.4. Dynamic light scattering, transmission electron microscopy and electron paramagnetic resonance techniques were used to characterize the average particle size, polydispersity, zeta potential, morphology and fluidity of the liposomes. *In vitro* dialysis experiments showed that the combined liposomal system provided significantly longer (72 h) release of ropivacaine, compared to conventional liposomes (~45 h), or plain ropivacaine (~4 h) (p <0.05). The pre-formulations tested were significantly less toxic to 3T3 cells, with toxicity increasing in the order: combined system < ropivacaine in donor or acceptor liposomes < ropivacaine in conventional liposomes < plain ropivacaine. The combined formulation, containing 2% ropivacaine, increased the anesthesia duration up to 9 h after subcutaneous infiltration in mice. In conclusion, a promising drug delivery system for ropivacaine was described, which can be loaded with large amounts of the anesthetic (2%), with reduced *in vitro* cytotoxicity and extended anesthesia time.

## Introduction

Local anesthetics (LAs) are small molecules that are rapidly removed from the site of administration, which limits the duration of anesthesia [1]. Ropivacaine (RVC) is an amino-amide local anesthetic that is widely used in surgical procedures. Its commercial form is the optically pure S(-) isomer, which has physicochemical and therapeutic properties comparable to those of bupivacaine. Advantages that justify the increasing clinical use of RVC include its lower cardiovascular and neurological toxicity, as well as greater selectivity for sensory rather than motor block in isolated nerves or epidural injection [2].

One approach to prolonging LA effect is to associate the molecules with carriers, such as liposomes, in order to retain them at the injection site for a longer time by means of a sustained release mechanism [3]. The gradual release of the anesthetic also helps to reduce its toxicity [4].

Liposomes are frequently used as carriers in drug delivery systems (DDS), and a number of liposome-based pharmaceutical products have been approved for clinical applications [5]. Due to their amphiphilic nature LAs interact with these biocompatible and biodegradable vesicles, and distribute themselves in the lipid bilayer region and in the aqueous core [6]. Several studies have described the prolongation of anesthetic effects after LA encapsulation in liposomes, in both animals and humans [4, 7]. In addition to the so-called conventional liposomes, new kinds of vesicles have been proposed to overcome limiting factors such as high serum levels/systemic toxicity (pegylated liposomes) [8], low skin penetration (elastic liposomes) [9, 10], low drug-to-lipid upload (ionic gradient liposomes) [11–13] and fast release (combined donor-acceptor liposomes) [14].

Transmembrane gradient liposomes (pH 7.4 outside/acidic pH inside the vesicles) have been described for the delivery of bupivacaine, the most common local anesthetic employed in surgery [7]. Both large unilamellar vesicles (LUV) [12] and large multivesicular vesicles (LMVV) [11] of different lipid compositions were able to incorporate up to 2% bupivacaine (compared to 0.5% of commercial solutions). The anesthetic could be actively (remotely) loaded into such vesicles because its uncharged form easily penetrated the lipid bilayer, acquiring a charge on arrival in the inner (acid pH) liposome compartment. In the ionic form, the LA was no longer able to cross the bilayer freely, so that the protonated species–of high aqueous solubility—prevailed in the acidic inner aqueous core of the liposomes, favoring the entrapment of large amounts of the anesthetic. The gradient liposomes provided higher encapsulation efficiency than conventional liposomes, and significantly prolonged the duration of anesthesia after intradermal administration in a human volunteer [11]. Moreover, in a patent of 2007 [14], Barenholz & Garbuzenko proposed the encapsulation of bupivacaine in a combined ionic-gradient liposomal system, comprising donor and acceptor LMVV. Donor liposomes were prepared with hydrogenated soy phosphatidylcholine (HSPC): cholesterol (3:2 mol %), and contained 250 mM (NH_4_)_2_SO_4_ and 4.5% bupivacaine in their inner aqueous core; acceptor vesicles were composed of dimyristoyl or dipalmitoyl-PC:cholesterol (3:2 mol %), also with 250 mM (NH_4_)_2_SO_4_ gradient. Acceptor liposomes were able to encapsulate the anesthetic released by the donor liposomes, causing bupivacaine to remain at the site of action for a longer period.

In this context, the objective of the present study was to prepare and characterize a new formulation for remote loading of RVC, based on donor-acceptor liposomes with transmembrane ionic gradients and to evaluate its *in vivo* anesthetic efficacy.

## Materials and methods

Hydrogenated soy phosphatidylcholine (HSPC) was purchased from Avanti Lipids Inc. (Alabaster, AL, USA). Cholesterol, 4-(2-hydroxyethyl)-1-piperazineethanesulfonic acid (Hepes), sodium acetate, Triton X-100, uranyl acetate and 5-SASL were purchased from Sigma Chem. Co. (St. Louis, MO, USA). Ammonium sulfate was acquired from Merck (São Paulo, SP, Brazil). Ropivacaine hydrochloride was kindly donated by Cristália Ind. Quím. Farm. Ltda (Itapira, SP, Brazil). Dulbecco’s Modified Eagle’s Medium (DMEM) was purchased from Nutricell (Campinas, SP, Brazil) and 3-(4,5-dimethylthiazol-2-yl)-2,5-diphenyltetrazolium bromide (MTT) was from Calbiochem Corp. (La Jolla, CA, USA).

### Preparation of conventional and ionic gradient liposomes

Dry lipid films containing HSPC and cholesterol (2:1 mol %) were prepared by solvent evaporation under a nitrogen flow. The last traces of solvent were removed under vacuum for at least 2 h. Large multilamellar vesicles were obtained by hydration of the lipid film with 250 mM ammonium sulfate in 50 mM Hepes buffer (pH 7.4) or 50 mM sodium acetate buffer (pH 5.5), up to a final lipid concentration of 15 mM (2:1 HSPC/cholesterol), confirmed by phosphate and cholesterol quantification assays. After vortexing for 10 min, the multilamellar vesicles were either (i) extruded through 400 nm pore size polycarbonate membranes (12 cycles, 40 psi, at 60°C) to form LUV, or (ii) extruded through 100 nm pore size membranes (Lipex Biomembranes Inc., Vancouver, BC, Canada), and then subjected to 10 freeze-thaw cycles (using liquid nitrogen and a water bath at 37°C), with agitation, to form LMVV [15]. The acceptor vesicles, with internal pH of 5.5 (denoted LUV 5.5_in_), were prepared with 50 mM acetate buffer at pH 5.5. The donor vesicles (denoted LMVV 7.4_in+ sulfate_) were prepared with 50 mM Hepes buffer (pH 7.4) plus 250 mM ammonium sulfate.

To create the ionic gradients, both types of liposomes were centrifuged twice at 120,000 × *g* for 2 h at 4°C [16]. The pellet was then suspended in 50 mM Hepes buffer so that the external pH of the liposomes was always equal to 7.4. One day before use, the donor liposomes were incubated with 0.75% or 2% RVC for 45 min at 60°C [11], agitated overnight at room temperature (SK 0330 Pro Shaker, Scilogex, Cambridge, UK), and then stored under refrigerated conditions until required. The combined liposomal system was prepared immediately prior to the experiments by adding the acceptor vesicles, stored under refrigeration, to the suspension of donor liposomes (1:1 v/v) under agitation [14]. The final formulation used in the experiments contained 15 mM total lipids and 0.75% or 2% RVC.

Formulations containing only the donor or the acceptor vesicles were also prepared for comparison. Other controls included conventional liposomes (LMVV 7.4_in_ and LUV 7.4_in_) prepared without any ionic gradient and from multilamellar vesicles (as described above) in 50 mM Hepes buffer (pH 7.4); when necessary, RVC was actively encapsulated into these conventional liposomes, as described above.

### Determination of liposome morphology

The morphology of the donor and acceptor vesicles was examined by transmission electron microscopy (TEM) using a Zeiss-LEO 906 microscope (Carl Zeiss Group, Oberkochen, Germany) operated at 80 kV. A 50 μL sample of the liposomal suspension was placed onto a copper grid (200 mesh) for 10 s. A 2% uranyl acetate solution was then dropped onto the grid, the excess volume was removed with filter paper, and the samples were dried by incubation at room temperature for 4 h. Such methodology has been previously adopted to check the morphology of other liposomal formulations [10, 15, 17]. ImageJ software (NIH, USA) was used for the statistical analysis of LMVV size distribution in TEM images.

### Determination of liposome size, polydispersity index (PDI) and zeta potential

The average particle size (nm), polydispersity index (PDI) and zeta potential (mV) of the liposomal formulations were determined before and after RVC encapsulation by dynamic light scattering, using a Zetasizer ZS-90 particle analyzer (Malvern Instruments, Malvern, UK). The samples, diluted in deionized water, were analyzed 24 h after preparation, at a fixed angle of 90° and at 25°C [17, 18].

### Encapsulation efficiency determination

The RVC encapsulation efficiency (%EE) of the suspensions of LMVV 7.4_in+ sulfate_ (or LUV 5.5_in_ loaded with RVC, only used in the characterization tests) was determined after ultrafiltration-centrifugation (4000 × *g* for 20 min) in a Millex 10 kDa regenerated cellulose filtration device (Millipore, Bedford, MA, USA). The RVC present in the filtrate was determined by high performance liquid chromatography (HPLC), using a Prostar 410 instrument (Varian, CA, USA), according to a validated method [15]. Samples were diluted (1:9) in 50 mM Hepes buffer (pH 7.4) and aliquots were injected onto a Purospher^®^ STAR RP-18E column (octadecylsilane, 150 x 4.6 mm; Merck KGaA, Darmstadt, Germany). The mobile phase was acetonitrile:phosphate buffer (60:40 v/v, pH 8.0), the flow rate was 1.2 mL/min, and the absorbance was measured at 240 nm [15]. Aliquots of liposomes without RVC were used as controls. The %EE was calculated using Eq 1:

$$\%\mathrm{EE}=\frac{{\text{RVC}}_{\text{trapped}}}{{\text{RVC}}_{\text{total}}}.100$$ (1)

where RVC_trapped_ refers to the RVC encapsulated in the liposomes, and RVC_total_ is the initial (total) amount of RVC added to the formulation. RVC_trapped_ was calculated as the difference between RVC_total_ and the amount of free RVC, measured by HPLC in the filtered fraction as described above.

### Lipid bilayer organization

The fluidity of the donor and acceptor liposomes was determined by electron paramagnetic resonance (EPR), as described previously [19]. The order parameters (S) of the liposome bilayers were determined from the spectra of the spin probe, 5-doxyl stearate (5-SASL), incorporated into the vesicles (LMVV 7.4_in_+sulfate plus LUV 5.5_in_) at up to 1 mol %, in the absence and presence of 2% RVC. The EPR spectra were recorded using a Bruker ELEXSYS spectrometer (Bruker BioSpin GmbH, Karlsruhe, Germany) operated at 9.7 GHz and room temperature (21 ± 1°C). The order parameter (S) for 5-SASL, whose long molecular axis is roughly parallel to the normal bilayer, was calculated according to Eq 2 [20]:

$$\text{S}=\frac{{\text{A}}_{//}\;\text{-}\;{\text{A}}_{┴}}{\left[\text{Azz-}\;(\text{Axx}+\text{Ayy})/2\right]}$$ (2)

where A// and A_┴_ are the hyperfine splitting of the spin label’s long molecular axis oriented parallel and perpendicular, respectively, to the external magnetic field.^37^ A_ZZ_ (32 Gauss), A_YY_ (6 Gauss), and A_XX_ (6 Gauss) are the values of the principal components of the hyperfine tensor, assuming a fully oriented sample [19].

### *In vitro* measurement of ropivacaine release

The *in vitro* release of RVC from the liposomes was monitored at 25°C using a dialysis system in which two compartments were separated by a cellulose membrane (MWCO 12000–14000 Da, Spectrapore). The upper compartment contained the test sample (1 mL of plain RVC, RVC in LMVV 7.4_in+ sulfate_, RVC in LUV 5.5_in_, or RVC in the combined liposome system) and the lower compartment was filled with 100 mL of 50 mM Hepes buffer (pH 7.4). Samples were withdrawn from the lower compartment at regular intervals for determination of RVC concentration by UV absorption at 260 nm [18].

KinetDS 3.0 software was employed to analyze the profile release curves [21], testing several kinetic models. The 0 order (Eq 3) and Weibull models (Eq 4) were the best fitting models considering the coefficient of determination (R^2^).

$${Q=\mathrm{k.t}+Q}_{0}$$ (3)

$$Q=1\text{-exp}\left[\frac{{\text{-}(\text{t})}^{\text{b}}}{\text{a}}\right]$$ (4)

where *Q* is the amount of RVC released at the time *t*, *Q*_0_ is the starting value of *Q*, k is the rate constant, *b* is the release exponent, and *a* is the timescale of release.

### Cell viability assay

The 3T3 cell line (perpetual Balb/c mouse fibroblasts) was obtained from NIH (National Institute of Health, Baltimore-USA). The cells were routinely grown in DMEM medium containing 10% fetal bovine serum, 100 U/mL penicillin, and 100 μg/mL streptomycin, at 37°C in a humidified incubator with 5% CO_2_. The cells (2 × 10^4^ per well) were incubated in 96-well plates for 48 h until semi-confluence. They were then treated for 2 h with 0.8, 3.2, 6.4, 8.0, 12.0 or 16.0 mM of RVC, either alone or associated with the different liposomal systems: LMVV 7.4_in+ sulfate_; LUV 5.5_in_; combined liposome system; conventional liposomes prepared with no salt (LMVV 7.4_in_) or proton (LUV 5.5_in_) gradients. RVC-free liposomes were used as experimental controls [18]. After treatment, the growth medium was replaced with MTT solution (1 mg/mL) and the cells were incubated for 1 h at 37°C. The MTT solution was then removed and ethanol (0.1 mL) was added to dissolve the formazan crystals. The formazan absorbance was measured at 570 nm using a microplate reader (ELx800, Bio Tek Instruments, Inc., Winooski, VT, USA). At least 5 experiments per treatment were conducted. The results (mean ± SD) were expressed as a percentage of the value obtained for untreated controls [22]. Fifty percent inhibitory concentration (IC_50_) values were determined by nonlinear regression analysis using a sigmoidal concentration-response equation based on individual experiments. This analysis was performed using Origin v.6.0 (Microcal Software, Inc., Northampton, MA, USA).

### Analgesia (von Frey) tests

The animal study was approved by the Ethics Committee on Animal Research (CEUA) of the State University of Campinas (protocol # 2595–1), which follows the Principles of Laboratory Animal Care (NIH publication #85–23, revised in 1985).

*In vivo* studies were performed in groups of 7 adult male *Mus musculus* mice (25-30g), obtained from CEMIB-UNICAMP (Centro Multidisciplinar para Investigação Biológica, University of Campinas, Brazil), certified by the International Council for Laboratory Animal Sciences (ICLAS). The animals (three per cage) underwent a period of acclimatization (12:12 h light-dark cycle, 23 ± 2°C, water and food *ad libitum*) for 7 days.

The paw withdrawal test was performed to evaluate the animal paw movement in response to pressure; a von Frey filament (Insight Equip., Rib. Preto, SP, Brazil) was applied perpendicularly to the plantar surface of the right hind paw [23]. Thirty minutes before testing, seven mice were placed in individual acrylic cages (11 x 9 x 7 cm) with a wire grid floor (0.5 x 5 cm). Baseline measurements (3 per animal) were then performed by applying pressure in the hind paw with the von Frey filament. The initial force applied to the animal's paw was 0.0073 N; this force was gradually increased to the maximum of 0.456 N or until the animal removed its paw [24]. A tilted mirror below the grid provided a clear view of the mice hind paw. Subsequently, 25 μL of each formulation (plain, donor, acceptor or combined liposomes) containing RVC (0.75% or 2.0%) was subcutaneously administered in the hind paw and measurements were made at 0, 30, 60, 90 and 120 min post-injection, and then at 60-min intervals until the response returned to predetermined baseline values. The same observer performed all experiments and the results were expressed as the percentage of animals with analgesia as a function of time. At the end of the study, the animals were sacrificed by deepening general anesthesia with Ketamine/Xylazine.

### Statistical analysis

The data were analyzed using Instat v.3 (GraphPad Software, Inc., San Diego, CA, USA). The unpaired t-test was used to evaluate the particle diameter, PDI, zeta potential, EPR and encapsulation efficiency data. One-way analysis of variance (ANOVA) with the post-hoc Tukey-Kramer multiple comparisons test was used to analyze the data on release kinetics and cellular viability, and results of analgesia tests. Statistical significance was indicated by a p-value of <0.05.

## Results and discussion

RVC is a good local anesthetic for use during surgical procedures and the post-operative period [25]. Use of liposomal drug delivery systems for the encapsulation of RVC has been reported previously [18, 26, 27]. Our research group has previously reported a 1.5-fold increase in the release time of RVC from large unilamellar liposomes composed of egg PC:cholesterol:α-tocopherol (4:3:0.07 mol %), compared to plain RVC [18]. Furthermore, in a recent investigation of ionic-gradient (pH and salt) liposomes, the established transmembrane gradient allowed active loading and retention of high amounts (2%) of RVC in the vesicles (multilamellar, LUV and LMVV of egg PC:cholesterol:α-tocopherol), prolonging the release time of the anesthetic for up to 25 h [15].

The use of gradient liposomes [28] in a combined manner, as donor-acceptor vesicles [14] was adopted in the present study to maximize the benefits of the RVC encapsulation by further increasing the release time and improving its pharmacological activity. Donor (large multivesicular liposomes containing 250 mM ammonium sulfate plus 2% RVC) and acceptor (large unilamellar vesicles, internal pH 5.5) vesicles were prepared with HSPC:cholesterol (2:1 mol %) at pH 7.4.

The combined liposomal formulation has the advantage of bringing together three innovative approaches (different lipid compositions, vesicle types and ionic-gradients) to prolong the effect of ropivacaine. Selection of these approaches for this study (to guarantee the suitable membrane fluidity to prolong the effect of ropivacaine) was based on our previous experience [4, 6, 15].

### Morphology of the vesicles

**Fig 1:**
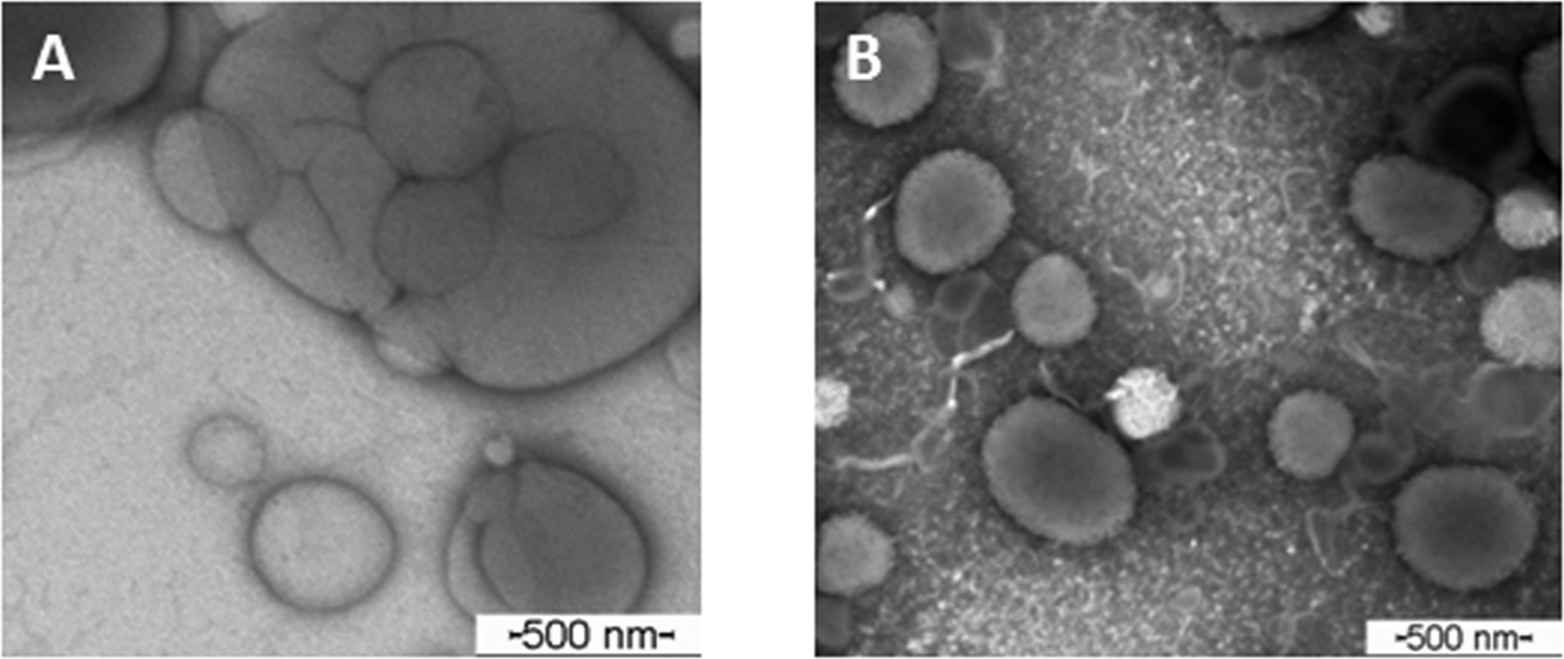
Transmission electron micrographs of HSPC:cholesterol (2:1 mol %) vesicles. A) donor—LMVV 7.4_in+ sulfate_ and B) acceptor—LUV 5.5_in_, stained with 2% uranyl acetate (× 60,000 magnification).

TEM images of the donor (LMVV) and acceptor (LUV) liposomes are shown in Fig 1A and 1B. The micrographs provide evidence that the methodology used to prepare the vesicles was satisfactory, showing clear differences between the large multivesicular (Fig 1A) and unilamellar (Fig 1B) vesicles. As expected, the LMVV were larger than the LUV, probably due to the freeze-thaw process used in their preparation. Due to the lack of homogeneity in donor liposome size and morphology, we additionally performed some quantitative analyzes on LMVV micrographs. Using the ImageJ software to analyze TEM micrographs (S1 Fig), we confirmed the spherical features and broad size distribution of the donor liposomes, compared to the LUV.

### Determination of liposome size, polydispersity index and zeta potential

Dynamic light scattering measurements were performed using the donor, acceptor, and combined liposomes. The data confirmed the morphology results (Fig 1), with the extruded (acceptor) vesicles presenting diameters near 400 nm (Table 1) and acceptable polydispersion (PDI ~0.2), whereas LMVV and the combined liposome systems presented larger average particle sizes (~1000 nm) and higher polydispersity than the LUV. While PDI is not a strong parameter to guarantee the homogeneity of the acceptor vesicles, and despite the inhomogeneity of LMVV particle sizes, previous studies have shown they were suitable for subcutaneous application [11, 29]. Moreover, a recent report describes the use of a depot formulation (proliposome RVC oil) that forms large multilamellar vesicles (1390 ± 580 nm), extending the pharmacokinetics of the anesthetic when subcutaneously applied in human volunteers [27]. Addition of RVC did not have a significant effect on the size or polidispersity of the vesicles, even the acceptor ones (Table 1) to which RVC was added in order to test the effect of the anesthetic on these parameters.

**Table 1:** Size, polydispersity index (PDI) and zeta potential of HSPC:cholesterol (2:1 mol %) liposomes.

	Without RVC			With 2% RVC		
Vesicles	Diameter (nm)	PDI	Zeta potential (mV)	Diameter (nm)	PDI	Zeta potential (mV)
**Donor**	900±30	0.7	-44.9±3.1	980±80	0.6	-39.7±4.7
**Acceptor**	538±1*	0.2	-1.0±0.1*	484±4	0.2	-14.8±0.2
**Combined**	1152±88	0.7	-11.9±0.3	1120±140	0.7	-13.1±0.8

Donor (LMVV 7.4_in+ sulfate_), acceptor (LUV 5.5_in_) liposomes and their combination (LMVV 7.4_in+ sulfate_ + LUV 5.5_in_), before and after encapsulation of 2% ropivacaine; N = 5.

* LUV (without RVC) vs. LUV with RVC; statistically significant difference (p<0.05); unpaired t-test.

For all formulations (Table 1) the zeta potentials were negative, an indication of physical stability due to charge repulsion on the surface of the donor vesicles [30], with the LMVV presenting the highest values. Zeta potentials remained the same or increased (in module) in the presence of RVC. Since RVC mainly exists in the charged form at pH 7.4, the maintenance of similar zeta potentials in the presence of RVC indicates that there are few anesthetic molecules at the surface of the vesicles. Instead, the majority of the anesthetic is in the inner aqueous compartments of the liposomes. The minor fraction present in the membrane phase is probably deeply inserted between the lipids, as shown previously for bupivacaine [31].

### Encapsulation efficiency (%EE)

Table 2 lists the %EE values for RVC in the donor and acceptor liposomes, as well as in the conventional liposomes prepared with no salt (LMVV) or proton (LUV) gradients. The conventional liposomes showed comparable encapsulation efficiency (24.3 ± 2.8% and 30.0 ± 6.6% for LMVV 7.4_in_ and LUV 7.4_in_, respectively), with no statistically significant difference between them. These results are in good agreement with our previous findings (23.8 ± 3.5% for LUV composed of egg PC:cholesterol:α-tocopherol; 4:3:0.07 mol %) [18].

**Table 2:** RVC encapsulation efficiency of HSPC:cholesterol (2:1 mol %) liposomes.

	Formulation	RVC encapsulation (%)
Donor	LMVV 7.4_in+ sulfate_	42.0±3.2*^a^
Acceptor	LUV 5.5_in_	72.1±4.8*^b^^,^ *^c^
Control donor (no gradient)	LMVV 7.4_in_	24.3±2.8
Control acceptor (no gradient)	LUV 7.4_in_	30.0±6.6

Statistically significant differences (p<0.05, unpaired t-test)

*^a^—in comparison to conventional multivesicular liposomes (LMVV 7.4_in_)

*^b^—in comparison to donor (LMVV 7.4_in+ sulfate_)

*^c^—in comparison to conventional unilamellar liposomes (LUV 7.4_in_).

Significant improvements in %EE were obtained for both gradient liposomes, with the LUV (acceptor) liposomes providing more effective encapsulation than the sulfate gradient LMVV (donor) liposomes (Table 2). Upload capacities of the donor and acceptor vesicles were increased by 75% and 140%, respectively, in comparison to conventional liposomes (without any gradient); drug/lipid molar ratios of 0.75 and 1.4 were achieved with LMVV and LUV, respectively. As the fraction of RVC inserted in the lipid bilayer should have been unaffected by the gradient, this result unequivocally proves the higher upload capacity of donor and acceptor vesicles due to formed ionic gradients, with the major part of the RVC molecules remaining in the aqueous core of the liposomes.

Since the donor vesicles possessed a salt and pH gradient (established by ammonium dissociation, NH_4_^+^ → NH_3_ + H^+^, and ammonia leakage from the vesicles), and the acceptor vesicles had a pH gradient alone as the driving force to entrap the protonated anesthetic species inside the liposomes [28], it was expected that the donor vesicles would provide more efficient encapsulation than the acceptor vesicles. However, acceptor vesicles proved to be more effective than the donor ones in terms of uploading the charged RVC species. A high upload capacity of the acceptor vesicles is desirable in order to ensure the encapsulation of RVC molecules released from the donor vesicles.

To understand that, we used the average sizes of LMVV and LUV (950nm and 500nm, respectively–see Table 1) to estimate the number of lipids/vesicle (e.g. 2.85 × 10^6^ for LUV) and the number of vesicles (3.16 × 10^15^ and 4.79 × 10^14^ for LUV and LMVV, respectively) in 1 L of the 15 mM liposome suspension. Calculation of the amount of lipids in the oligolamellar liposomes was based on each spherical LMVV having three internal lamellae of 500 nm diameter. In addition, the trapped volumes of LUV and LMVV were estimated as 13.8 and 14.3 μl/μmol lipid, respectively, corresponding to *ca*. 20% of the total formulation volume. Neither the (similar) trapped volumes of LUV and LMVV, nor the higher magnitude of the LMVV over the LUV ionic gradient could account for the higher encapsulation of RVC in the LUV. Thus, vesicle type and the higher number of acceptor vesicles (*ca*. 7-fold greater than that of donor vesicles) appear to explain their higher %EE and potential to remote load the RVC molecules released from the donor liposomes. So, the single bilayer of LUV, as opposed to the oligolamellar “barriers” of LMVV, seems to favor RVC encapsulation in the acceptor vesicles.

### Lipid bilayer organization

EPR is a useful technique for the assessment of changes in lipid bilayer organization caused by the insertion of amphiphilic molecules between the lipids [6].

**Fig 2:**
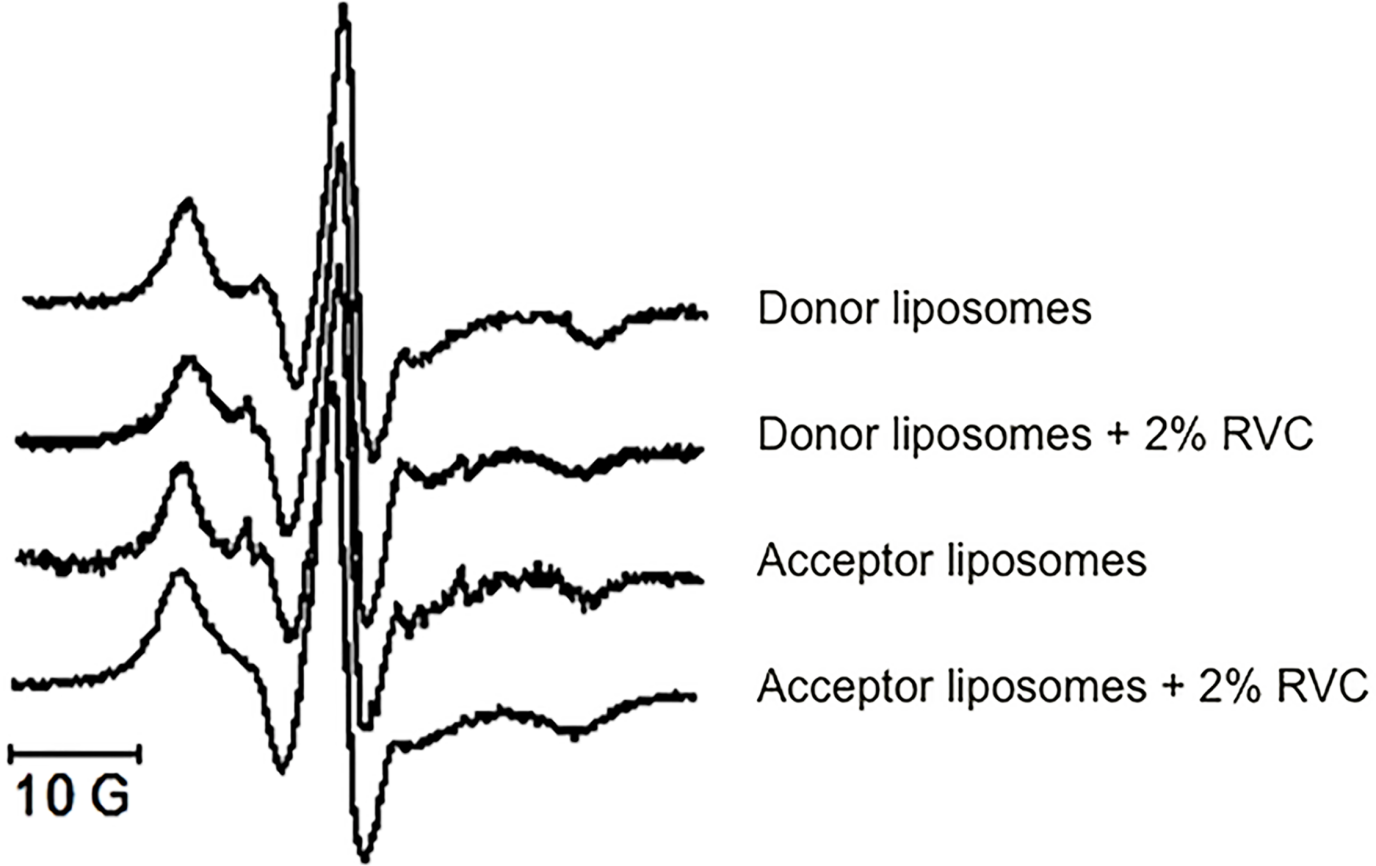
EPR spectra of 5-SASL probe incorporated in liposomes, with or without 2% RVC: donor (LMVV 7.4_in+ sulfate_) and acceptor (LUV 5.5_in_) vesicles.

Fig 2 shows the EPR spectra of 5-SASL inserted as a probe to monitor the lipid environments of the donor and acceptor liposomes. The nitroxide spectra revealed a very immobilized signal, indicative of a high level of lipid packing in the HSPC:cholesterol vesicles.

The order parameter (S) values determined from the spectra of 5-SASL incorporated into the vesicles, before the incorporation of RVC, were 0.83 ± 0.02 and 0.84 ± 0.01 for the donor and acceptor liposomes, respectively. The values of S were similar for the LMVVs and LUVs, revealing that the preparation process gave rise to vesicles of similar fluidity. Addition of RVC to the vesicles slightly decreased the S values of donor vesicles to 0.79 ± 0.01 (p <0.05; unpaired t-test, LMVV with *vs*. LMVV without RVC), and of acceptor vesicles to 0.82 ± 0.03. These results are consistent with the well-known disorganizing effect of local anesthetics on membranes [6].

In addition to the similar fluidity of donor and acceptor vesicles, EPR results revealed the compactness of the HSPC:cholesterol liposomes, with S >0.8. This value corresponds to a high (4) lipid rigidity, according to Zucker and coworkers [32]. In research previously conducted in our laboratory, the S value measured using 5-SASL inserted into multilamellar egg PC vesicles was 0.65 [6], while a value of 0.78 was obtained for LUV composed of egg PC:cholesterol (3:2 mol %) at ambient temperature [19]. Even in the case of biological (erythrocyte) membranes, which are more organized because of their protein content, the order parameter value (0.76) measured under the same conditions [33] did not surpass that of the HSPC:cholesterol liposomes reported here. A high level of lipid packing was therefore an important factor contributing to the sustained release of the anesthetic from the vesicles [14, 33].

EPR results confirmed the proposed mechanism for the sustained release of the anesthetic from the combined system, involving ionization and partition of the anesthetic from donor to acceptor vesicles. Briefly, the charged RVC encapsulated by the donor liposomes: (1) slowly deionizes and crosses the membrane to join the free RVC molecules in solution, at pH 7.4; (2) but a fraction of the RVC molecules in solution partition into the acceptor liposomes, whose inner acidic pH favors its ionization and entrapment; (3) with different kinetics, the RVC fractions (1 + 2 + 3 –see Graphical Abstract) can reach the nervous membrane, 2 being faster (guaranteeing the onset of anesthesia), and 1 and 3 being responsible for the prolonged effects observed (see below).

### *In vitro* release kinetics

**Fig 3:**
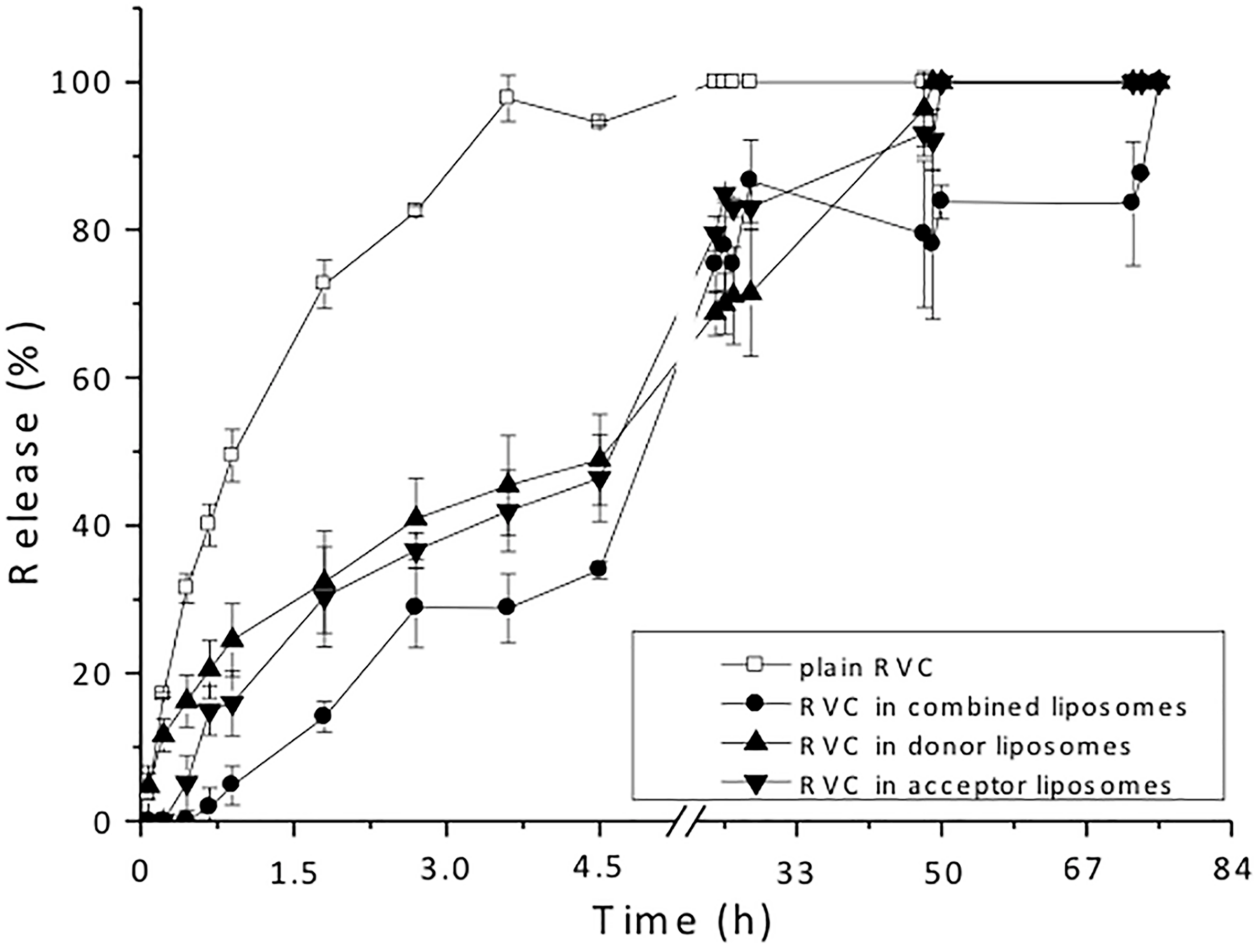
*In vitro* release kinetics of RVC, plain or encapsulated in HSPC:cholesterol liposomes: donors (LMVV 7.4_in+ sulfate_), acceptors (LUV 5.5_in_), and their combination (LMVV 7.4_in+ sulfate_ + LUV 5.5_in_); Ambient temperature (25°C), N = 3.

The hyperbolic curve in Fig 3 revealed that 100% release of unencapsulated RVC (plain RVC) occurred after 4 h. The equilibrium time observed for RVC in the gradient liposomes was extended to around 50 h (both for donor and acceptor liposomes) and as long as 72 h for the combined formulation, revealing a remarkable 18-fold increase in the sustained release of the anesthetic (see also S1 Table).

Similar results were reported by Grant et al. [11] for the release of bupivacaine from LMVV of the same lipid composition: while *ca*. 75% of the drug was released from the liposomes after 24 h at 37°C, only 10% was set free when the experiment was carried out at ambient temperature (21°C). The prolonged *in vitro* release promoted by the two types of liposomes (and their combination) in the present study was probably related to the higher degree of packing of the bilayer composed of HSPC:cholesterol (see EPR results), since faster complete release of RVC from egg PC:cholesterol:α-tocopherol unilamellar liposomes (7 h) has been reported [18].

Mathematical modeling is a powerful means of describing the release profile of DDS [34]. Using the KinetD 3.0 software, the RVC release curves from the donor, acceptor and combined liposomes were fitted using several models. Among them, the zero-order model was the best fit found to describe the curves of RVC release from donor and acceptor liposomes, as shown in S2 Table. As for the combined liposomes, the best fit was achieved with the Weibull model (S2 Table), which has been previously used to describe the kinetic curves of other liposomal formulations [35, 36]. In that case, the release exponent from Eq 4 (b value, S2 Table) was greater than unity (sigmoid curve), indicating a complex release mechanism [37]. These results reveal that, while diffusion can approximately describe the release of RVC from donor and acceptor vesicles, more than one mechanism affects the delivery of RVC from the combined liposome formulation, accounting for its prolonged RVC release profile. Similar results were described by Barenholz and Garbuzenko [12], who demonstrated that bupivacaine molecules released by the donor liposomes were encapsulated again by the acceptors, delaying the drug release and metabolism in comparison to conventional liposomes [14].

More importantly, the kinetic studies revealed the capacity of the combined liposomes to provide extended sustained release of RVC. The sustained release attained with combined liposomes indicates that the anesthetic will reside at the site of action, increasing the duration of anesthesia, as observed before with conventional liposomes [18].

### Cell viability assay

**Fig 4:**
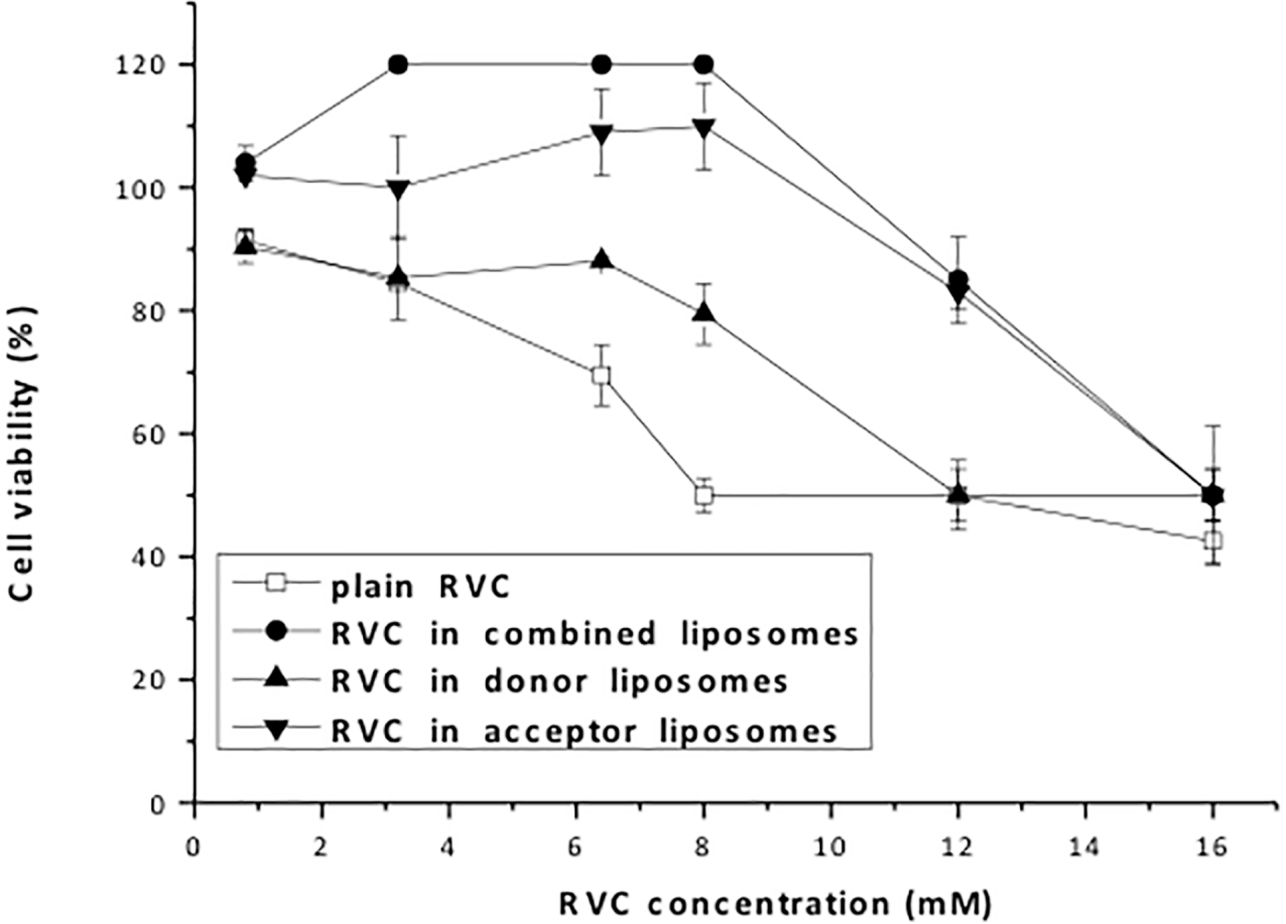
Cell viability (%) of 3T3 fibroblasts exposed to RVC, plain or encapsulated in liposomes: donors (LMVV 7.4_in+ sulfate_), acceptors (LUV 5.5_in_), and their combination (LMVV 7.4_in+ sulfate_ + LUV 5.5_in_); N = 5.

The presence of RVC substantially reduced the viability of 3T3 fibroblast cells in culture (Fig 4), in agreement with our previous results using sciatic nerve Schwann cells [18]. RVC and other local anesthetics are cytotoxic to cells due to mechanisms related to their membrane binding, such as the inhibition of mitochondrial enzymes [38] or cell lysis [39]. S2 Fig shows the curves obtained with the control liposomes (donor, acceptor and combined) that were found to be no toxic to the cells.

Encapsulation in liposomes is known to reduce the cytotoxicity of anesthetics [40]. In this study, encapsulation of RVC in the liposomes significantly diminished its cytotoxicity (IC_50_ = 8mM for plain RVC), with the combined formulation (RVC in LMVV 7.4_in+ sulfate_ + LUV 5.5_in_) presenting lower cytotoxicity (IC_50_ = 16mM) than the donor, acceptor, and conventional liposomes containing RVC (Fig 4). The control liposomes—without RVC—were not toxic to 3T3 cells, as previously shown [18]. The decrease in toxicity was consistent with the extended sustained release profile obtained for the combined liposomes (Fig 3), as reported above.

Barenholz & Garbuzenko similarly reported that the use of a combined gradient liposomal formulation resulted in a 8-fold increase in the LD_50_ value of bupivacaine, intraperitoneally injected in mice [14]. Even though these authors employed different lipids (HSPC:cholesterol and dimyristoyl or dipalmitoyl-PC:cholesterol for donor and acceptor LMVV, respectively), the results presented here show that liposomes with the same composition (HSPC:cholesterol) and similar membrane fluidity, but different types of vesicles (LMVV and LUV), are able to reduce RVC cytotoxicity when used in combination.

### Antinociceptive tests

To assess the anesthetic effect, the paw withdrawal test was performed in mice using the von Frey filament, with formulations containing 0.75% and 2% RVC. The anesthesia curves representing the percentage of mice that were anesthetized are presented in S3 Fig. The effect was dose-dependent and RVC in combined liposomes showed increased anesthesia times compared to formulations containing the donor or acceptor liposomes separately, or compared to plain RVC. Table 3 shows the statistical analysis, taking into consideration the area under the curves for S3 Fig.

**Table 3:** Analgesia (von Frey test) induced by ropivacaine in mice. The anesthetic was used in solution (0.75% and 2% plain RVC) or encapsulated in HSPC:cholesterol (2:1 mol %) liposomes: donor (RVC in LMVV 7.4_in+ sulfate_), acceptor (RVC in LUV 5.5 _in_) and combined formulation (RVC in LMVV 7.4_in+ sulfate_ + LUV 5.5_in_).

Formulation	AUC	Analgesia time (h)
0.75% plain RVC	13.66 ± 3.71	4
0.75% RVC in donor liposomes	28.81 ± 5.83	6
0.75% RVC in acceptor liposomes	16.58 ± 3.72	6
0.75% RVC in combined liposomes	43.48 ± 6.77*^a^^,^ *^b^^,^ *^c^	7
2% RVC plain RVC	13.92 ± 2.96	5
2% in donor liposomes	31.08 ± 6.14*^d^	7
2% in acceptor liposomes	17.76 ± 2.89*^e^	7
2% RVC in combined liposomes	45.58 ± 7.26*^f^^,^ *^g^^,^ *^h^	9

AUC = area under the curve. Statistically significant differences (two-way ANOVA/Tukey test; *p<0.05) in groups of 7 animals each

*^a^ Combined (0.75% RVC in LMVV 7.4_in + sulfate_ + LUV 5.5_in_) vs. 0.75% plain RVC

*^b^ Combined (0.75% RVC in LMVV 7.4_in + sulfate_ + LUV 5.5_in_) vs. acceptor (0.75% RVC in LUV 5.5_in_)

*^c^ Combined (0.75% RVC in LMVV 7.4_in + sulfate_ + LUV 5.5_in_) vs. donor (0.75% RVC in LMVV 7.4_in + sulfate_)

*^d^ Donor (2% RVC in LMVV 7.4_in + sulfate_) vs. 2% plain RVC

*^e^ Acceptor (2% RVC in LUV 5.5_in_) vs. 2% plain RVC

*^f^ Combined (2% RVC in LMVV 7.4_in + sulfate_ + LUV 5.5_in_) vs. 2% plain RVC

*^g^ Combined (2% RVC in LMVV 7.4_in + sulfate_ + LUV 5.5_in_) vs. acceptor (2% RVC in LUV 5.5_in_)

*^h^ Combined (2% RVC in LMVV 7.4_in + sulfate_ + LUV 5.5_in_) vs. donor (2% RVC in LMVV 7.4_in + sulfate_).

The combined liposomal formulation containing 0.75% RVC induced the longest analgesia (*ca*. 7 h), followed by donor or acceptor liposomes (*ca*. 6 h); analgesia with 0.75% plain RVC was *ca*. 4 h. When RVC was applied at 2% (Table 3, S3B Fig), even longer analgesia times were achieved: *ca*. 9 h for the combined liposomes, followed by the donor or acceptor liposomes (*ca*. 7 h each) and 2% plain RVC (*ca*. 5 h).

Previous studies have reported long-lasting anesthesia attained with ionic gradient liposomes using bupivacaine [12, 29]. To our knowledge, this is the first *in vivo* study to employ ionic gradient liposomes to deliver RVC. Our results show a 1.8-fold increase in the analgesia duration after use of the combined (ionic gradient) liposomal formulation, in comparison to 2% plain RVC. The increase in analgesia time *in vivo* was not as marked as might be expected based on results of the *in vitro* RVC release experiments (Fig 3). However, it is important to note that the *in vitro* experiments were conducted at lower temperature (25°C), compared to 37°C for *in vivo* experiments and when higher rates of RVC clearance and metabolism can be anticipated. This temperature difference may explain the apparent discrepancy between the *in vitro* and *in vivo* results.

## Conclusions

The development of a combined liposomal system composed of two types of vesicles with transmembrane ionic gradients successfully overcame the drawbacks of conventional liposomal formulations, such as reduced encapsulation efficiency and fast drug release of local anesthetics. Ionic gradient liposomes were effective in entrapping the ionized RVC species inside the vesicles, while the combined donor-acceptor system ensured the sustained release of the anesthetic. Although there are published reports of other liposomal formulations being used for the delivery of RVC [18, 27], to our knowledge, this is the first report of a pharmaceutical formulation able to effectively upload large amounts of RVC (2%) and afford sustained *in vitro* release, while presenting lower cytotoxicity and a significant improvement in the sensory block after subcutaneous infiltration.

Avoiding cardiac toxicity is a challenge in peri-operative anesthesia using long-acting anesthetic agents. Even enantiomeric pure LAs of lesser toxicity, such as RVC and levobupivacaine, have been shown to cause systemic effects [41]. In this scenario, the formulation presented here shows a promising future in long surgical procedures, and post-operative or chronic pain treatment.

## Supporting information

S1 Fig LMVV micrographs (× 60,000 magnification) and statistical analysis of particle size distribution data, obtained with ImageJ software. (TIF)

S2 Fig Cell viability (%) of 3T3 fibroblasts exposed to control (without ropivacaine) liposomes: donors (LMVV 7.4_in+ sulfate_), acceptors (LUV 5.5_in_), and their combination (LMVV 7.4_in+ sulfate_ + LUV 5.5_in_); N = 5. (TIF)

S3 Fig Analgesia (von Frey) tests. A) 0.75% and B) 2% ropivacaine, plain or encapsulated in liposomes: donors (LMVV 7.4_in+ sulfate_), acceptors (LUV 5.5_in_), and the combined system; N = 7 mice per group.
(TIF)

S1 Table Statistical analysis of ropivacaine release from solution (plain RVC) and HSPC:cholesterol (2:1 mol %) liposomes. (DOCX)

S2 Table Fitting of the kinetic data of RVC release with different mathematical models. (DOCX)

## Abbreviations

DMEM: Dulbecco’s Modified Eagle Medium
DDS: drug delivery systems
EPR: electron paramagnetic resonance
Hepes: 4-(2-hydroxyethyl)-1-piperazineethanesulfonic acid
HPLC: High performance liquid chromatography
HSPC: Hydrogenated soy phosphatidylcholine
IC_50_: 50% inhibitory concentration
LA: Local anesthetics
LD_50_: 50% lethal dose
LMVV: Large multivesicular vesicles
LMVV 7.4_in_: Conventional LMVV liposomes
LMVV 7.4_in+ sulfate_: Donor liposomes
LUV: Unilamellar vesicles
LUV 5.5_in_: Acceptor vesicles with internal pH of 5.5
LUV 7.4_in_: Conventional LUV liposomes
MTT: 3-(4,5-dimethylthiazol-2-yl)-2,5-diphenyltetrazolium bromide
PC: phosphatidylcholine
PDI: Polydispersity index
RVC: Ropivacaine
RVC_total_: Initial (total) amount of RVC added to the formulation
RVC_trapped_: RVC encapsulated in the liposomes
S: order parameter
TEM: Transmission electron microscopy
%EE: Encapsulation efficiency
3T3: perpetual Balb/c mouse fibroblasts cell line
5-SASL: 5-doxyl stearate spin probe
A// and A_┴_: Hyperfine splitting in EPR spectra
