# Supplementary figures and images for: Encapsulation of ropivacaine in a combined (donor-acceptor, ionic-gradient) liposomal system promotes extended anesthesia time
Source: PLoS One. 2017 Oct 5;12(10):e0185828. doi: 10.1371/journal.pone.0185828 (PMC5628885; doi:10.1371/journal.pone.0185828)

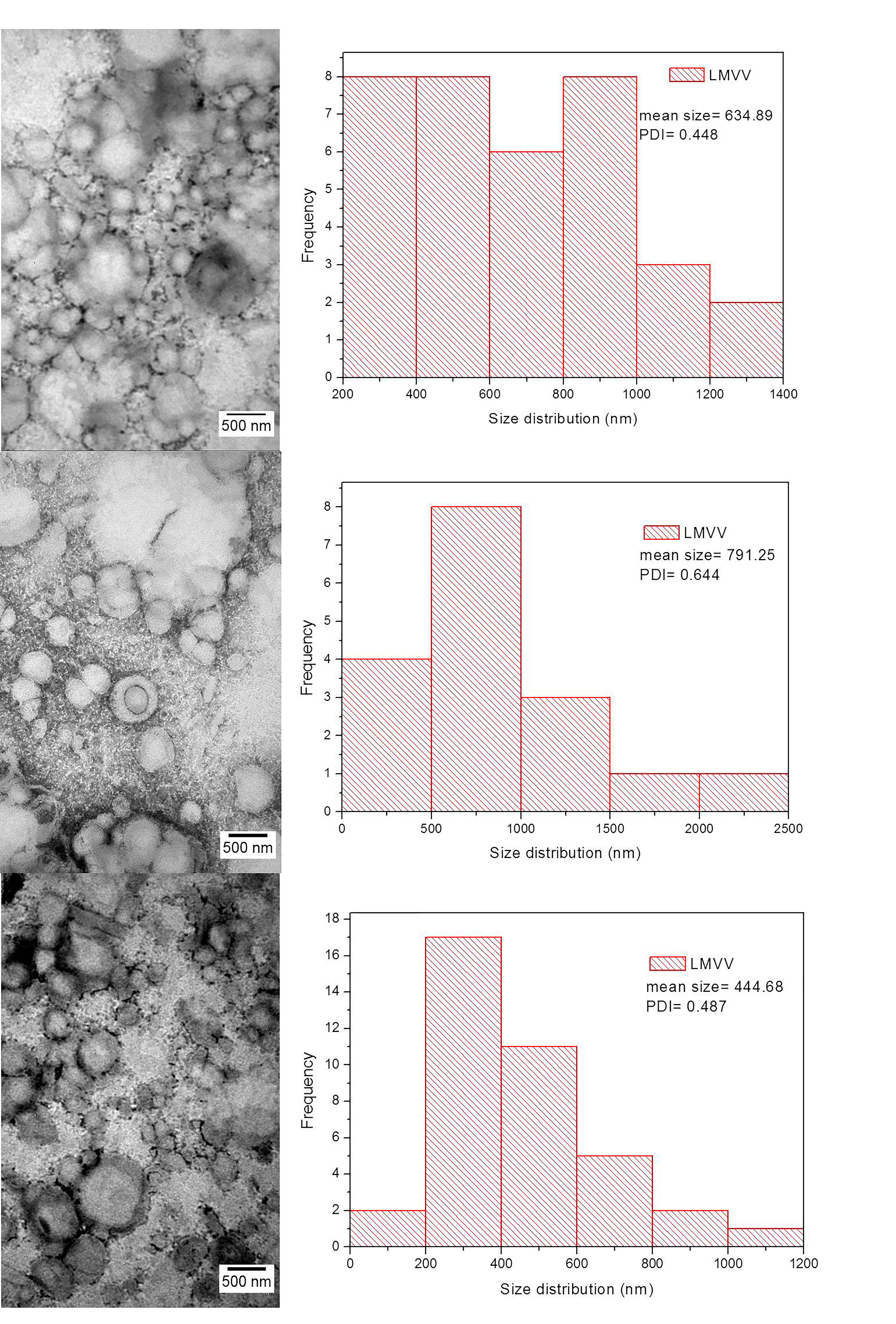

Supplement: S1 Fig — (TIF) [file pone.0185828.s001.tif]

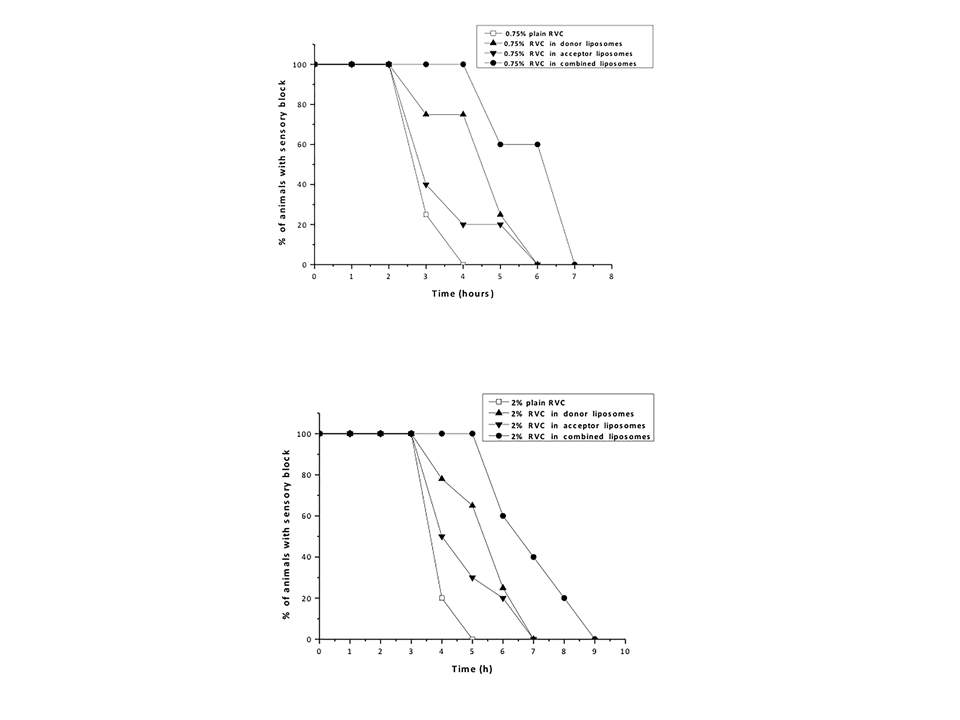

Supplement: S3 Fig — A) 0.75% and B) 2% ropivacaine, plain or encapsulated in liposomes: donors (LMVV 7.4in+ sulfate), acceptors (LUV 5.5in), and the combined system; N = 7 mice per group. (TIF) [file pone.0185828.s003.tif]
