# Supplementary material for: Encapsulation of ropivacaine in a combined (donor-acceptor, ionic-gradient) liposomal system promotes extended anesthesia time
Source: PLoS One. 2017 Oct 5;12(10):e0185828. doi: 10.1371/journal.pone.0185828 (PMC5628885; doi:10.1371/journal.pone.0185828)
Supplement: S1 Table — (DOCX) [file pone.0185828.s004.docx]

# SUPPORTING INFORMATION

**S1 Table**: **Statistical analysis of ropivacaine release from solution (plain RVC) and HSPC:cholesterol (2:1 mol %) liposomes**.

| **Formulation** | **50% Release time (h)** | **100% Release time (h)** | **AUC ± SD**  **(100%)** |
| --- | --- | --- | --- |
| Plain RVC | 1.0 | 4.0 | 32841.1 ± 129.5 |
| RVC in donor liposomes | 5.8 | 49.0 | 235785.5 ± 4532.1*^a^ |
| RVC in acceptor liposomes | 7.0 | 50.0 | 241522.7 ± 8017.0*^b^ |
| RVC in combined liposomes | 12.3 | 72.0 | 438034.7 ±432.5*^c,d,e^ |

Donor (LMVV 7.4_in+ sulfate_), acceptor (LUV 5.5_in_), and combined (LMVV 7.4_in+ sulfate_ + LUV 5.5_in_) liposomes. AUC = area under the curve.

Statistically significant differences (one-way ANOVA/Tukey-Kramer test; *p<0.05):

^*a^ Donor liposomes (RVC in LMVV 7.4_in+ sulfate_) versus plain RVC;

^*b^ Acceptor liposomes (RVC in LUV 5.5_in_) versus plain RVC;

^*c^ Combined liposomes (RVC in LMVV 7.4_in+ sulfate_ + LUV 5.5_in_) versus plain RVC;

^*d^ Combined versus acceptor liposomes (RVC in LUV 5.5_in_);

^*e^ Combined versus donor liposomes (RVC in LMVV 7.4_in+ sulfate_).
