# Supplementary material for: Encapsulation of ropivacaine in a combined (donor-acceptor, ionic-gradient) liposomal system promotes extended anesthesia time
Source: PLoS One. 2017 Oct 5;12(10):e0185828. doi: 10.1371/journal.pone.0185828 (PMC5628885; doi:10.1371/journal.pone.0185828)
Supplement: S2 Table — (DOCX) [file pone.0185828.s005.docx]

**S2 Table: Fitting of the kinetic data of RVC release with different mathematical models**.

| **Model** | **R^2^** | **a** | **b** | **Liposome formulations** |
| --- | --- | --- | --- | --- |
|  | 0.77 | 1.29 | 2.60 | Acceptor |
| Zero order | 0.85 | 1.20 | 2.88 | Donor |
|  | 0.76 | 1.28 | 1.80 | Combined |
|  | 0.52 | 2.26 | -6.05 | Acceptor |
| Weibull | 0.81 | 0.80 | -1.38 | Donor |
|  | 0.91 | 1.26 | 3.76 | Combined |

R^2^ is the coefficient of determination. **Z**ero order model: **a** and **b** refer to the slope and intercept, respectively of the linear fit (equation 3). Weibull model: **a** and **b** are defined in equation 4.
